# Key demographics and psychological skills associated with adjustment to progressive Multiple Sclerosis early in the diagnosis

**DOI:** 10.3389/fresc.2022.966133

**Published:** 2022-08-29

**Authors:** Angeliki Bogosian, Fern Day, Sam Norton, Eli Silber, Mohamed Sakel, Basil Sharrack, Rona Moss-Morris

**Affiliations:** ^1^ School of Health and Psychological Sciences, University of London, London, United Kingdom; ^2^ Department of Psychology, Institute of Psychiatry, Psychology & Neuroscience, King's College London, London, United Kingdom; ^3^ Department of Neurology, King’s College Hospital, London, United Kingdom; ^4^ Department of Neurorehabilitation, East Kent Hospitals University NHS Foundation Trust, Kent, United Kingdom; ^5^ Department of Neuroscience and NIHR Neurosciences Biomedical Research Centre, Sheffield Teaching Hospitals NHS Foundation Trust and University of Sheffield, Sheffield, United Kingdom

**Keywords:** multiple sclerosis, progressive, psychosocial factors, adjustment, model, quantitative research, management

## Abstract

**Background/purpose:**

Being diagnosed with a progressive type of multiple sclerosis (MS) has been associated with worse psychological outcomes compared to relapsing-remitting type. Previous studies of adjustment to MS have primarily focused on relapsing-remitting type MS. The present study aims to examine psychological adjustment for people newly diagnosed with progressive multiple sclerosis.

**Methods:**

This was a multicenter cross-sectional survey of 189 people newly diagnosed with progressive MS. A composite measure of psychological adjustment was created from questionnaires measuring psychological distress, positive affect, perceived-stress, life satisfaction and self-concept. Predictor variables included coping strategies, social support, relationship with partner, psychological vulnerability, MS-related beliefs, and responses to symptoms. Data were analysed using a regularised regression model to indicate which group of all variables are associated with adjustment.

**Results:**

People who were older (*b* = 0.17(0.07), *p* = 0.02), in employment (*b* = 0.40 (0.17), *p* = 0.01), and with lower illness severity (*b* = −0.24 (0.08), *p* = 0.001) showed better adjustment. Based on a Lasso regression, the most important psychological and demographic variables associated with lower adjustment (out-of-sample cross-validation *R*^2 ^= 62.6%) were lower MS self-efficacy and higher avoidance, cognitive vulnerability, embarrassment avoidance, conflict, helplessness, and secondary progressive MS type.

**Conclusions and implications:**

Helping newly diagnosed people to find ways to tolerate anxiety-causing situations by encouraging acceptance may help people adjust to progressive MS by lowering their avoidance. Further, building confidence in managing the illness and addressing relationship issues are key focus areas in psychological interventions for people with progressive multiple sclerosis.

## Introduction

Multiple sclerosis (MS) is a chronic disease of the central nervous system that affects more than 2.5 million people worldwide, including around 127,000 people in the United Kingdom (1). It usually strikes during early adulthood and can lead to disabling symptoms across many body systems (motor, sensory, gastrointestinal, genitourinary, visual, etc.) and marked impairments in psychological, social, cognitive, and occupational function. There are three forms of MS. Relapsing-remitting MS is the most common form and is characterised by random attacks that can leave permanent deficits, followed by periods of remission during which people may feel quite well (2). About 10–15 per cent of MS cases have a progressive course from the outset (known as primary progressive MS, PPMS), which involves a continuing deterioration in symptoms and increase in disability without remission. Most people with relapsing-remitting MS will eventually transition to the progressive form of MS (secondary progressive MS, SPMS) (3).

Psychological adjustment promotes physical and mental health outcomes of people with MS (4). A 2020 meta-review showed that people with MS experience psychological stress, worries about diagnosis and prognosis, challenges faced during the diagnostic process, and disruption of everyday life functions and roles (5). This is not surprising when considering that individuals with MS deal with a wide range of challenging symptoms including pain and fatigue, increasing disability including for some loss of mobility, as well as uncertainties about their future physical capacities, ambiguities about the future course of their illness, and concerns about their ability to resume their former lifestyle. How individuals contend with these issues may influence current and future psychological and physical adaptation to MS.

Adjustment is a complex, multi-faceted construct that is defined and measured in different ways in the literature, making comparisons of results among studies problematic (6). Distress, self-concept, functional impairment, and quality of life, for example, are all adjustment outcomes measured in previous studies; however, these capture only certain aspects of adjustment and thus provide an incomplete picture of the underlying adjustment construct. A recent systematic review showed that people with MS use emotional and avoidance coping strategies more than other types of coping, particularly in the early stages of the condition (7). “Coping” is a broad concept made up of several distinct types of coping, some of which may include behaviours also captured by measurements of “responses to MS” and “social support”. Finally, there is a preponderance of research in people with relapsing-remitting MS, which may not reflect the experiences of people with progressive forms of MS who tend to experience a poorer quality of life and face greater ongoing adjustment challenges as their illness progresses (6).

Several frameworks and theoretical models have been used to understand adjustment and guide interventions in MS (5). These models suggest factors predicting adjustment to MS and are either specifically developed for the context of MS (8–10) or were adapted models from the wider literature (11–13). These models were based on research that mainly included people with relapsing-remitting MS or mixed samples of the MS population, so the relevance of all the predicting factors to people with progressive MS who may face different challenges and symptoms is not clear.

We aim to address such methodological issues and the gap in the literature regarding adjustment in progressive MS by measuring multiple adjustment indicators and psychosocial factors in the same individuals who have progressive MS. This work will allow us to explore psychosocial factors important to people’s adjustment. This study will allow us to:

1. To describe the type and severity of psychological challenges experienced by people with PPMS and SPMS early on in diagnosis.
2. To explore the overlapping variance of key outcome measures in progressive MS.
3. To determine the contribution of demographics, psychological, social and other environmental factors to individuals' adjustment to progressive MS.

The answers to these questions will help us identify those most likely to struggle to adjust to their illness and understand which factors are associated with less successful adjustment. This knowledge will enable the effective targeting of interventions to those at the most significant risk of struggling to adapt to their illness and experiencing long-term impaired wellbeing. Further, knowing the factors more relevant for adjustment to progressive MS will help us identify the most relevant psychological treatment for this group.

## Participants and methods

Before commencing the project, the Wales Research Ethics Committee 7 (16/WA/0034) and City University Psychology Research Ethics Committee (PSYETH (S/F) 15/16 101) approved the study.

### Sample size

We used an *a priori* sample size calculator for structural equation models (14), where this model evaluated the associations between the seven psychological adjustment measures. Specifying a conservative anticipated effect size of 0.14, seven latent variables, 36 observed variables, 80% power, and 0.05 probability level. The power calculation indicated a minimum sample size of 109 to (a) allow estimation given the complexity of the model structure and 290 to (b) detect the effect between latent variables.

### Participant selection

We recruited participants through a mixture of rural and urban MS clinics (*n* = 30) throughout the UK and advertisements on the MS Society website. Clinical staff in participating clinics identified potential participants from the clinic case list who met the study inclusion criteria. Eligible patients were approached about the study by clinical staff. All people who were interested in participating, including those recruited *via* advertisements, were given a Participant Information Sheet to read about the study and had the opportunity to ask a researcher questions about taking part.

### Exclusion criteria

People with recent onset primary progressive or secondary progressive multiple sclerosis were eligible for participation in the study provided they did not meet any of the exclusion criteria. The exclusion criteria for the study were as follows:

▪ People under the age of 18 years
▪ People with relapsing-remitting or benign MS
▪ People diagnosed with primary progressive MS for more than five years
▪ People who transitioned to secondary progressive MS more than five years ago
▪ People who had limited or no ability to speak English

### Measurements

A systematic review by Dennison et al. (6), grouped psychosocial variables into “over-arching conceptually or thematically related categories” which, although measured differently between studies, were found to be related to adjustment. We mapped the variables in these studies into these broad overarching themes identified previously in the literature. We then reviewed a large selection of questionnaires with good psychometric properties that have previously been used to measure adjustment and psychosocial factors in people with MS. A preliminary selection of questionnaires was reviewed by two project consultants who have progressive MS who provided feedback about the instruments and their experience of completing a large battery of questionnaires which was used to refine the selection to the final measures presented below.

We used five measures of seven constructs to define psychological adjustment that were previously used in the MS literature. These measures included:

1. Psychological distress (Hospital Anxiety and Depression Scale; (15)). The HADS measures symptoms of anxiety (7 items) and depression (7 items). The total score for each subscale ranges from 0 to 63, with 0–9 considering no anxiety/ depression, 10–18, mild to moderate anxiety/depression and 19–29 moderate to severe anxiety and 30–63 severe anxiety.
2. Positive affect (Positive and Negative Affect Schedule; (16)). The PANAS measures positive affect (10 items) and negative affect (10 items). The total score for each subscale ranges from 10 to 50, with higher scores indicating higher positive and negative affect.
3. Life Satisfaction (Satisfaction with Life Scale; (17)). The Satisfaction with Life Scale was developed to assess satisfaction with people’s lives. The scale does not assess satisfaction with specific life domains, such as health or finances, but allows subjects to integrate and weigh these domains in whatever way they choose. The possible range of scores is 5–35, with a score of 20 representing a neutral point on the scale. Scores between 5 and 9 indicate the respondent is extremely dissatisfied with life, whereas scores between 31 and 35 indicate the respondent is extremely satisfied.
4. Self-concept (Self-Concept Questionnaire; (18)). The Self-Concept Questionnaire consists of 30 statements relating to different areas of self-concept. The scores range from 0–210, with higher score means higher sense of self-concept.
5. Perceived stress (Perceived Stress Scale-10; (19)). The PSS measures the degree to which situations of one's life are appraised as stressful and distressing. Items were designed to tap how unpredictable, uncontrollable and overloaded respondent find their lives. The scores range from 0–40 with higher scores indicating higher perceived stress.

Factors influencing adjustment

1. Length of illness
2. Severity of symptoms and mobility (Expanded Disability Status Scale Self-Report; (20))
3. Participant background (Social-demographic Questionnaire)- employment
4. Coping (Coping Strategy Indicator; CSI), (21)). The CSI measures situational coping encompassing the strategies of problem-solving (11 items), seeking social support (11 items) and avoidance (11 items). The scores for each subscale range from 0–22, higher scores indicate greater use of the strategy.
5. Social support (Interpersonal Relationship Inventory; (22)). The IPRI measures three key dimensions of interpersonal relationships: (1) perceived social support (13 items), (2) perceived conflict (13 items), and (3) perceived reciprocity (13 items). Scores on each subscale can range from 13 to 65, with higher scores indicating greater perceived social support, conflict and reciprocity.
6. Relationship with a partner (Dyadic Adjustment Scale; (23)). The DAS assess the relationship quality of intact (married or cohabiting) couples. The total score rage from 0 to 151, with higher scores indicating more positive dyadic adjustment.
7. Psychological vulnerability (Psychological Vulnerability Scale; (24)). The PVS scale measures psychological vulnerability related to perceptions of dependency, perfectionism, negative attributions, and the need for external sources of approval, all these cognitions can make people more susceptible to stress. The scores range from 6 to 30, with higher scores indicating higher psychological vulnerability.
8. MS-related cognitions – acceptance, helplessness, perceived benefits (Illness Cognitions Questionnaire; (25)). ICQ measures illness beliefs and consists of 3 subscales: helplessness (6 items), acceptance (6 items), and perceived benefits (6 items). The score ranges from 18 to 72, with higher scores indicating stronger presence of illness cognition.
9. Understanding of MS (Brief Illness Perception Questionnaire; (26)). The BIPQ is a 9-item questionnaire designed to rapidly assess cognitive (consequences, timeline, personal control, treatment control, identity, coherence) and emotional representations of illness. The BIPQ uses a single-item approach to assess perception on a 0–10 response scale, with higher scores indicating more threatening view of the illness. To minimise participants' burden we chose to include only items that were previously shown to be consistently associated with adjustment in MS, i.e. consequences, personal control, coherence and emotional representation.
10. Responses to symptoms (Cognitive & Behavioural Responses to Symptoms Questionnaire; (27)). The scale includes five cognitive subscales; fear avoidance, embarrassment avoidance (6 items), catastrophising about symptoms (4 items), beliefs that symptoms signal damage to the body (damage beliefs) (7 items), and symptom focus (6 items). There are also two behavioural subscales; resting and avoidance of activity (8 items) and all-or-nothing behaviour (5 items). All items are scored on a five-point frequency scale ranging from never (0) to all the time (4). Higher scores indicate more unhelpful responses. After consultation with people with progressive MS on the questionnaire pack, we excluded the catastrophising about symptoms, beliefs that symptoms signal damage to the body and symptom focus subscales of this questionnaire, as it felt they were not appropriate in the context of progressive MS.
11. MS Self-efficacy (Multiple Sclerosis Self-efficacy Scale; (28)). Self-efficacy is the subjective belief that one can overcome challenges that one is faced with. This is a 14-item scale with scores ranging from 14 to 84, with higher scoring indicates higher self-efficacy.

### Statistical analyses

To address objective 1, we used bivariate correlations, two-sample *t*-tests, Pearson's chi-squared and Wilcoxon rank-sum to examine relationships between psychological variables and type of MS and other participants' demographic and disease characteristics. Principal components analysis was used to evaluate objective 2 and estimate the shared variance between the seven adjustment measures (HADS-A, HADS-D, PANAS-NA, PANAS-PA, PSS10, CSQ) and test whether a one-factor unidimensional structure with a single general adjustment variable was acceptable using parallel analysis (29) After confirming a one-factor solution was optimal, confirmatory factor analysis using full-information maximum likelihood estimation was used to estimate the factor loadings and scores on the latent psychological adjustment factor for each participant. To address, objective 3, hierarchical linear regression analyses were conducted to determine demographics, psychological, and social factors drawn from the adjustment in progressive MS model (30) predicted adjustment. Separate regression models were estimated for each factor adjusting for age, gender and MS type. This was followed by analysis of all factors in a regularised regression model, specifically a least absolute shrinkage and selection operator (lasso, (31)), which was used to identify the subset of factors explaining the greatest amount of variance in psychological adjustment. This approach is related to stepwise selection of variables but avoids several of the associated problems. Importantly for the present study, shrinkage of the coefficients avoids their overestimation and provides more appropriate (i.e. generalisable) predictions where multiple correlated variables are included in the model

## Results

Two hundred and twenty-seven people completed the study questionnaires. Of the 406 patients with a neurologist confirmed MS diagnosis approached in NHS clinics, 215 (53%) completed the postal or online questionnaire. Twelve (5%) participants from the MS Society UK website responded. Informed consent and completed questionnaires were obtained during the period from January 2016 to August 2017.

Of the 227 participants who completed the questionnaires, 32 were excluded from the analysis (7 reported having RRMS and 38 reported having “other/unknown” type of MS), leaving 189 participants with primary or secondary progressive MS. Table 1 shows the disease and demographic characteristics of the progressive MS sample (*n* = 189). Participants were between 27 and 81 years old (mean = 56.2 (9). Half the sample (106, 56.1%) were female and most of the sample were in a relationship (144, 80.4%) and had stopped or reduced work due to MS (141, 75.0%). Interestingly, symptom severity and mobility issues, measured by EDSS, were greater in SPMS despite no difference in symptom change between the groups in the last year.

**Table 1:** Demographics and illness characteristics.

	Total	Primary progressive	Secondary progressive	*p*-value
	*N* = 189	*N* = 71	*N* = 118	
Age, years Mean (SD)	56.2 (9.0)	57.8 (10.3)	55.3 (8.1)	0.077
Female gender *N* (%)	106 (56.1%)	28 (39.4%)	78 (66.1%)	<0.001
Black, Asian and minority ethnic *N* (%)	13 (6.9%)	5 (7.0%)	8 (6.8%)	0.960
Lives alone *N* (%)	25 (13.3%)	13 (18.6%)	12 (10.2%)	0.100
In a relationship *N* (%)	144 (80.4%)	52 (76.5%)	92 (82.9%)	0.290
Low education *N* (%)	65 (34.6%)	24 (34.3%)	41 (34.7%)	0.950
In paid employment *N* (%)	45 (23.9%)	19 (26.8%)	26 (22.2%)	0.480
Stopped or reduced work due to MS *N* (%)	141 (75.0%)	43 (60.6%)	98 (83.8%)	<0.001
MS subtype, initial *N* (%) Primary progressive	75 (39.7%)	71 (100.0%)	4 (3.4%)	<0.001
Secondary progressive	13 (6.9%)	0 (0.0%)	13 (11.0%)	
Relapsing-remitting	84 (44.4%)	0 (0.0%)	84 (71.2%)	
Other/unknown	17 (9.0%)	0 (0.0%)	17 (14.4%)	
Time since diagnosis, years Mean (SD)	11.0 (5.0–21.0)	5.0 (3.0–-9.0)	16.0 (8.5–24.0)	<0.001
EDSS Mean (SD)	5.9 (1.4)	5.4 (1.5)	6.1 (1.2)	0.001
Symptom change in last year Mean (SD)	3.2 (0.8)	3.1 (0.8)	3.2 (0.9)	0.530

### Psychological challenges experienced by people newly diagnosed with primary progressive and secondary progressive MS

As shown in Table 2, on average participants' anxiety score was 7.4 (4.2), which reflects non-case of clinical anxiety and participants' depression score was 7.8 (4.0), which reflects non-case of clinical depression. Twelve (16.9%) of the 71 participants with primary progressive MS reported a depression score of over 10, indicating clinical depression, and eleven (15.5%) of the 71 participants with primary progressive MS reported an anxiety score of over 10, indicating clinical anxiety. Thirty-two (27.1%) of the 118 participants with secondary progressive MS reported a depression score of over 10, indicating clinical depression, and thirty-six (30.5%) of the 118 participants reported an anxiety score of over 10, indicating clinical anxiety. Initial t-tests demonstrated that people with secondary progressive MS reported experiencing higher levels of perceived stress, greater negative affect, a less positive self-concept and less satisfaction with life than those with primary progressive MS.

**Table 2:** Descriptive statistics of psychological adjustment variables.

	Total	Primary progressive	Secondary progressive	*p*-value
Psychological adjustment (overall score)	0.0 (1.0)	0.3 (1.0)	−0.2 (0.9)	0.001
Anxiety (HADS)	7.4 (4.2)	6.1 (3.8)	8.2 (4.2)	0.001
Depression (HADS)	7.8 (4.0)	6.8 (3.8)	8.4 (4.0)	0.006
Psychological distress (HADS total)	15.2 (7.0)	12.9 (6.6)	16.6 (7.0)	0.001
Positive affect (PANAS)	27.9 (8.4)	29.4 (9.3)	27.0 (7.7)	0.053
Negative affect (PANAS)	15.7 (8.2)	14.0 (7.9)	16.8 (8.2)	0.025
Satisfaction with Life Scale	16.7 (8.1)	18.3 (7.9)	15.8 (8.0)	0.037
Perceived stress (PSS)	18.3 (7.3)	16.5 (7.4)	19.4 (7.1)	0.009
Self-Concept Questionnaire	124.2 (29.9)	133.3 (30.1)	118.8 (28.6)	0.001

Comparing mean scores of psychological processes that are potentially contributing to psychological adjustment (table 3) shows that people with secondary progressive MS reported higher psychological vulnerability (*p* = 0.024), embarrassment avoidance (*p* = 0.015), all or nothing behaviour (*p* = 0.023) and MS-related self-efficacy (*p* = 0.014) compared to people with primary progressive MS.

**Table 3:** Descriptive statistics of psychological factors contributing to psychological adjustment.

	Total Mean (SD)	Primary progressive Mean (SD)	Secondary progressive Mean (SD)	*p*-value Mean (SD)
Problem solving (CSI)	24.0 (5.5)	23.4 (5.7)	24.3 (5.4)	0.32
Seeking social support (CSI)	20.7 (5.5)	20.3 (5.5)	20.9 (5.5)	0.51
Avoidance (CSI)	18.7 (4.9)	17.6 (4.9)	19.3 (4.7)	0.03
Reciprocity (IPRI)	49.4 (6.7)	50.4 (6.5)	48.8 (6.8)	0.11
Conflict (IPRI)	31.2 (9.2)	29.8 (8.3)	32.0 (9.7)	0.12
Relationship with partner (DAS)	17.6 (7.3)	16.9 (8.0)	18.0 (6.7)	0.35
Psychological vulnerability (PVS)	14.7 (5.6)	13.5 (5.5)	15.4 (5.6)	0.02
Helplesness (ICQ)	15.7 (4.1)	15.3 (4.2)	15.9 (4.0)	0.33
Acceptance (ICQ)	15.4 (3.8)	16.1 (3.8)	15.0 (3.8)	0.09
Perceive benefits (ICQ)	15.0 (4.3)	14.4 (4.5)	15.4 (4.1)	0.11
Consequences (BIPQ)	7.1 (2.2)	6.8 (2.4)	7.3 (2.2)	0.15
Personal control (BIPQ)	3.5 (2.5)	3.3 (2.5)	3.7 (2.5)	0.35
Coherence (BIPQ)	7.2 (2.3)	7.2 (2.3)	7.2 (2.4)	0.91
Emotional representation (BIPQ)	5.7 (2.6)	5.3 (2.6)	6.0 (2.5)	0.07
Embarrassment avoidance (CBRQ)	2.7 (1.1)	2.4 (1.1)	2.9 (1.1)	0.02
Avoidance/resting behaviour (CBRQ)	2.6 (0.7)	2.5 (0.8)	2.7 (0.7)	0.06
All-or-nothing behaviour (CBRQ)	2.9 (1.0)	2.6 (0.9)	3.0 (1.1)	0.02
MS self-efficacy	44.9 (11.1)	42.4 (11.5)	46.5 (10.6)	0.01

SD, Standard deviation.

### Defining psychological adjustment and the measures that best operationalise adjustment

**Figure 1:**
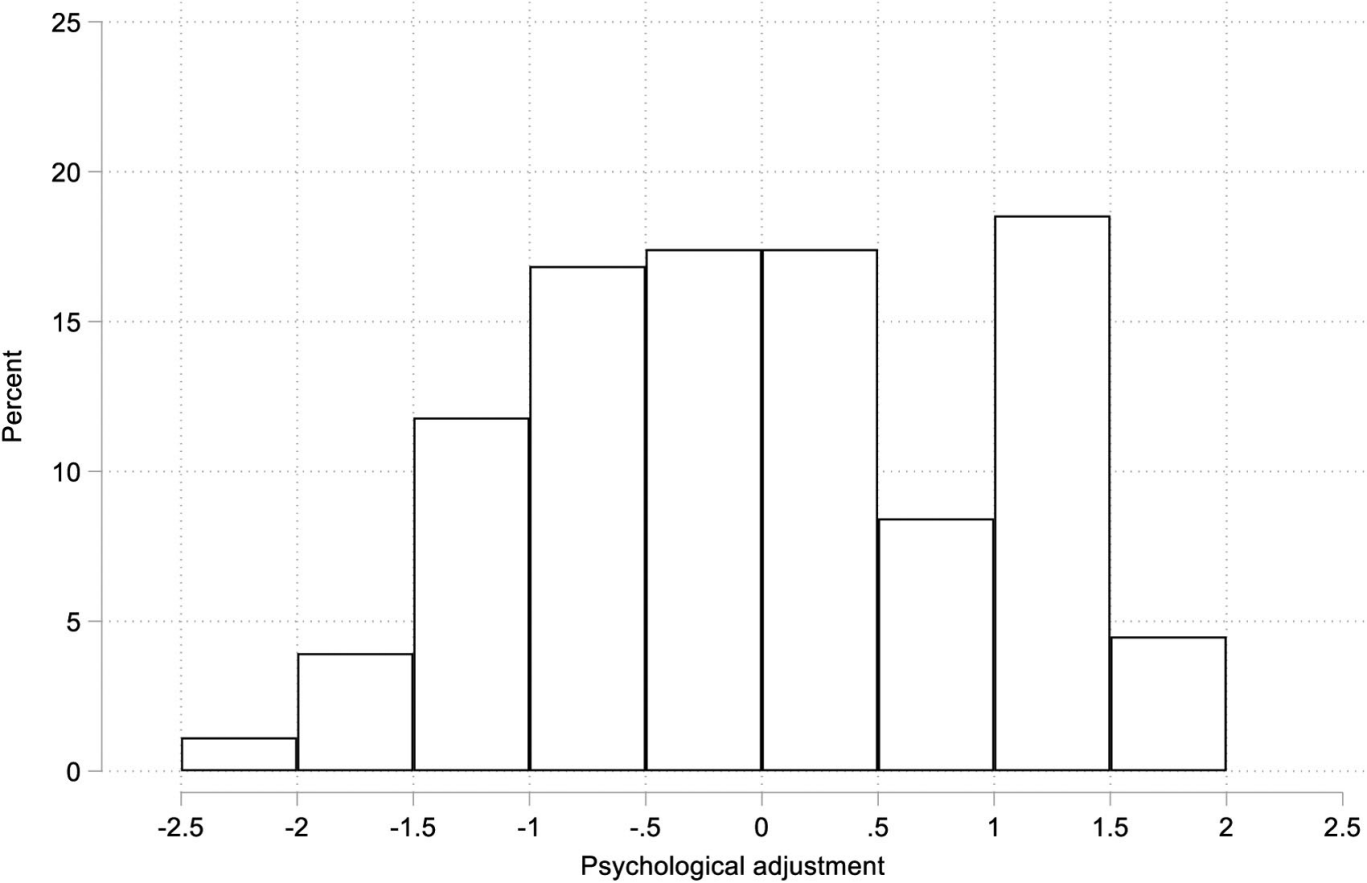
Distribution of general psychological adjustment variable (z-score) de estimated from a maximum likelihood confirmatory factor model.

The questionnaires that measure psychological distress (Hospital Anxiety and Depression Scale), positive affect (Positive and Negative Affect Schedule), perceived stress (Perceived Stress Scale-10), life satisfaction (Satisfaction with Life Scale) and self-concept (Self-concept questionnaire) were included in a confirmatory factor analysis model to estimate an overall psychological adjustment variable. Prior to this, a principal component analysis was undertaken, which confirmed a unidimensional structure, with the first component explaining 68% of the co-variance across the instruments and parallel analysis indicating only 1 underlying latent factor (see **Supplementary file**). The plot in Figure 1 shows the distribution of the factor score estimated from a maximum likelihood confirmatory factor model with a general psychological adjustment variable (RMSEA = .11; CFI = .97; TLI = .97. Chronbach’s alpha = .78). Here higher scores mean worse adjustment. The distribution is approximately normally distributed thought with a suggestion of a distinct particularly well-adjusted group score −1 or less (-1 means 1 standard deviation lower than the mean).

### Psychological factors contributing to psychological adjustment

Overall, demographics were not significantly correlated with psychological variables measured (see **Supplementary file 1**). The dyadic adjustment was positively correlated with living alone and negatively correlated with being in a relationship. The consequences illness perception was significantly correlated with stopping or reducing work due to MS, EDSS that measured illness severity was positively correlated with helplessness and illness consequences. The table in the **Supplementary file 1** shows that most of the psychological variables measured were significantly associated with the psychological adjustment factor and with each other.

Regularised regression, specifically a least absolute shrinkage and selection operator (lasso), was used to identify the subset of factors explaining the greatest amount of variance in psychological adjustment. In the first step, self-efficacy was selected as the variable explaining the largest amount of the variability in the data (29% variance explained), followed by psychological vulnerability, emotional response to illness, embarrassment avoidance, avoidance coping, social reciprocity, social support, being in paid employment, change in symptoms, dyadic adjustment, avoidance/resting behaviour, and being female. The final model explained 77% of the variance in psychological adjustment. **Supplementary file 2** shows data from the adjusted regression model for the adjustment score (HADS, PANA, PSS, life satisfaction & self-concept) regressed on all potential predictor variables, controlling for age, gender and MS type.

## Discussion

Progressive MS poses unique physical and psychological challenges for people's overall psychological adjustment, yet it is a less researched area. Investigating a large number of demographics and psychological variables from MS literature and variables identified in our qualitative work (30) and comparing them at the same time, gave us a good picture of psychological processes early on in the diagnosis of progressive MS. With the emergence of avoidance, cognitive vulnerability and self-efficacy as important factors, there is a clear direction and focus for future interventions. The findings of this study can also help clinicians identify the people who are most likely to struggle in adjusting to their illness (i.e. people with SPMS, people who have reduced or lost their job due to MS). This knowledge will enable the effective targeting of interventions to those who are at the greatest risk of struggling to adapt to their illness.

This study aimed to explore the overlapping variance of key outcome measures in progressive MS. Quantitative studies in MS have traditionally defined adjustment as psychological wellbeing (32–34), quality of life (32, 35) or the subjective impact of the illness on life domains (33, 36); however, these capture only certain aspects of adjustment and thus provide an incomplete picture of the underlying adjustment construct. The results of this study suggest that anxiety, depression, positive and negative emotions, perceived stress, satisfaction with life and self-concept all constructs contribute to the overarching concept of psychological adjustment. Future studies could examine whether we can develop a composite measure that includes all these variables to best operationalise the concept of adjustment to long-term condition. A longitudinal study could also show how fluid adjustment is and how much it changes overtime.

We found avoidance and psychological vulnerability to be key barriers and self-efficacy to be a key facilitator to adjustment. Avoidance coping refers to choosing your behaviour based on trying to avoid or escape thoughts or feelings. Previous studies also showed that emotional and avoidance coping strategies were used more than other types of coping especially in early stages of MS (7) and that avoidance and emotion-focused coping strategies were predictive of depressive symptoms and anxiety symptoms in those newly diagnosed with MS (37). Psychological vulnerability refers to core beliefs and thoughts that people have that can interfere with their daily life and negatively affect their mood. Previous studies showed psychological vulnerability mediating the relationship between adverse life events and MS symptoms (38). Interestingly, the results of this study showed that people with SPMS showed greater negative core beliefs compared to people with PPMS, which may indicate that the secondary progressive course of disease may make these core beliefs more salient.

Both avoidance coping and psychological vulnerability for people with progressive MS could be addressed through Acceptance and Commitment Therapy (39). In an Acceptance and Commitment approach there is a recognition that thoughts about MS may be realistic, for example, thoughts about progression and worsening of symptoms and the goal is to minimise their influence over people's lives. Under these principles, people are taught skills to step back from thoughts and take a distanced perspective on distressing content, even if that content is “true”.

Further, Acceptance Commitment Therapy focuses on facilitating acceptance and value-based actions (39) and that could be useful to address issues around avoidance of activities and embarrassment avoidance. Acceptance could be considered a feature of improving adjustment and quality of life (40). As shown in other studies in MS, increasing acceptance and decreasing avoidance of embarrassment would improve adjustment to MS (41). Acceptance is an alternative to our instinct to avoid thinking about negative or potentially negative experiences. It is the active choice to allow unpleasant experiences to exist without denying or changing them. Encouraging acceptance can be a way of encouraging action that will lead to minimizing avoidance. There are some preliminary evidence from small randomised control trials in MS, showing that Acceptance and Commitment Therapy can improve meaning of life variables (42), reduce anxiety (43), increase illness acceptance (44), and improve quality of life (45).

Self-efficacy was also associated with adjustment, as reflected in previous literature. A recent systematic review of 106 papers on MS showed that among other factors, higher self-efficacy was a protective factor for quality of life (46). The results are also in line with the self-efficacy theory (47) which indicates an association of self-efficacy with affective outcomes and research in MS showing links between higher reported self-efficacy and improved MS management (48) and lower reported disability (49). Supporting people to enhance their sense of capability to respond to MS challenges as well as teaching people necessary skills that could facilitate their sense of empowerment could be beneficial. Several psychological interventions have shown improvements in self-efficacy for people with MS. Emotional intelligence training where people with MS were supported to identify their emotions and then use emotions for problem-solving has been found to increase self-efficacy (50), as well as social cognitive training (51), progressive muscle relaxation (52), and a creative art programme (53).

The finding of the current study showed that relationship conflict can negatively affect adjustment. Previous studies have also shown that people with relapsing-remitting MS may have better social support than people with progressive MS (54) and the higher social support was associated with higher quality of life and lower anxiety (55) in people with MS. Couple's therapy could be a viable option as well as programmes to enhance social support. For example, a relationship enrichment programme significantly improved relationship satisfaction in couples living with MS, as well as improved mental health-related quality of life, communication, conflict resolution and ability to handle MS-specific functioning (56).

This study showed that people who had recently transitioned to SPMS might be more at risk of developing psychological issues. Previously, qualitative studies have underlined psychological and physical challenges that people with SPMS face (57). Jones et al. (58) studied the responses of 4178 people with MS on the MS UK register and found that people with SPMS were significantly more likely to be depressed than those with other types of MS. In a meta-ethnographic synthesis of qualitative studies showed that accepting and adapting coping strategies and the availability of social support and relationships promoted successful adjustment when transitioning to secondary progressive MS (59). There is some preliminary evidence showing the need expressed by people with SPMS for psychological support and a personalised care plan (60), as well as some preliminary evidence of the effectiveness of a computer-based cognitive neurorehabilitation (61). More research is needed to understand the specific challenges around SPMS and exploring potential support that could be offered around the transition period.

The cross-sectional nature of this study limits causal interpretation of the relationships between self-reported illness severity and other psychological factors. Another limitation is that all disease factors were collected *via* self-report instruments (including MS subtype, progression level, EDSS scores, and disease duration), which may be susceptible to either exaggeration or under-reporting by participants, where a clinician rating would have provided greater accuracy. Thirdly, the importance of non-significant psychological factors within our model may also be underestimated due to common-method variance and conceptual overlap with other psychological factors. Finally, even though we included a wide range of measures to capture aspects of psychological adjustment and psychological factors influencing adjustment, we have not included all possible variables. Our choice of questionnaires for the study reflected a balance between maximizing the number of potentially relevant psychosocial variables and minimizing the burden on participants. Where several instruments existed measuring the same variables, we prioritised the briefest with acceptable psychometric properties and those which had been completed by people with MS previously. This approach meant that some potentially important variables were not investigated in the study. Resilience, for example, is a relevant factor we did not explore which has been shown to influence psychological adjustment in MS (38) and would have been a valuable element in our study. Despite the limitations, this study reflects the largest progressive MS sample to date investigating a variety of potentially modifiable psychosocial factors, providing support for new treatment approaches in adjustment in progressive MS.

## Data Availability

The raw data supporting the conclusions of this article will be made available by the authors, without undue reservation.
