# Supplementary material for: Key demographics and psychological skills associated with adjustment to progressive Multiple Sclerosis early in the diagnosis
Source: Front Rehabil Sci. 2022 Aug 29;3:966133. doi: 10.3389/fresc.2022.966133 (PMC9583665; doi:10.3389/fresc.2022.966133)
Supplement: Supplementary file 1 [file Table_1.docx]

Supplementary file 1

Correlations among psychological variables measured and psychological adjustment factor
