# Supplementary material for: Key demographics and psychological skills associated with adjustment to progressive Multiple Sclerosis early in the diagnosis
Source: Front Rehabil Sci. 2022 Aug 29;3:966133. doi: 10.3389/fresc.2022.966133 (PMC9583665; doi:10.3389/fresc.2022.966133)
Supplement: Supplementary file 2 [file Table_2.docx]

Supplementary file 2

Regressions for the adjustment factor score on all potential predictor variables.

| Predictor variables | b | se | z | p | lci | UCI |
| --- | --- | --- | --- | --- | --- | --- |
| Age, years | 0.17 | 0.073 | 2.40 | 0.016 | 0.03 | 0.31 |
| Female gender | -0.07 | 0.15 | -0.48 | 0.630 | -0.36 | 0.21 |
| BAME | -0.32 | 0.29 | -1.09 | 0.277 | -0.90 | 0.26 |
| Lives alone | 0.02 | 0.21 | 0.07 | 0.941 | -0.40 | 0.43 |
| In a relationship | 0.12 | 0.18 | 0.65 | 0.513 | -0.24 | 0.48 |
| Low education | -0.02 | 0.15 | -0.16 | 0.875 | -0.31 | 0.26 |
| In paid employment | 0.41 | 0.17 | 2.43 | 0.015 | 0.08 | 0.73 |
| Stopped or reduced work due to | -0.21 | 0.17 | -1.23 | 0.217 | -0.54 | 0.12 |
| MS subtype, current | -0.37 | 0.14 | -2.64 | 0.008 | -0.65 | -0.10 |
| Time since diagnosis, years | 0.05 | 0.09 | 0.59 | 0.556 | -0.12 | 0.22 |
| LESS | -0.24 | 0.08 | -2.97 | 0.003 | -0.39 | -0.08 |
| Symptom change in last year | -0.15 | 0.07 | -2.19 | 0.029 | -0.29 | -0.02 |
| Problem solving (CSI) | 0.11 | 0.08 | 1.40 | 0.161 | -0.04 | 0.26 |
| Seeking social support (CSI) | 0.05 | 0.08 | 0.76 | 0.450 | -0.08 | 0.19 |
| Avoidance (CSI) | -0.57 | 0.06 | -9.43 | 0.001 | -0.68 | -0.45 |
| Reciprocity (IPRI) | 0.42 | 0.06 | 6.60 | 0.001 | 0.29 | 0.54 |
| Conflict (IPRI) | 0.44 | 0.06 | 6.88 | 0.001 | 0.31 | 0.56 |
| Relationship with partner (DAS) | -0.40 | 0.07 | -6.01 | 0.001 | -0.53 | -0.27 |
| Psychological vulnerability (PVS) | -0.30 | 0.07 | -4.18 | 0.001 | -0.44 | -0.16 |
| Helplessness (ICQ) | -0.56 | 0.06 | -9.50 | 0.001 | -0.68 | -0.45 |
| Acceptance (ICQ) | -0.59 | 0.06 | -9.96 | 0.001 | -0.71 | -0.47 |
| Perceive benefits (ICQ) | 0.51 | 0.06 | 8.16 | 0.001 | 0.39 | 0.63 |
| Consequences (BIPQ) | 0.32 | 0.07 | 4.66 | 0.001 | 0.18 | 0.45 |
| Personal control (BIPQ) | -0.39 | 0.07 | -5.95 | 0.001 | -0.52 | -0.26 |
| Coherence (BIPQ) | 0.26 | 0.07 | 3.79 | 0.001 | 0.13 | 0.40 |
| Emotional representation (BIPQ) | 0.07 | 0.07 | 0.96 | 0.001 | -0.07 | 0.21 |
| Embarrassment avoidance (CBRQ) | -0.59 | 0.06 | -9.98 | 0.001 | -0.70 | -0.47 |
| Avoidance/resting behaviour (CBRQ) | -0.63 | 0.06 | -11.12 | 0.001 | -0.74 | -0.52 |
| All-or-nothing behaviour (CBRQ) | -0.29 | 0.07 | -4.10 | 0.001 | -0.43 | -0.15 |
| MS self-efficacy | -0.20 | 0.07 | -2.83 | 0.005 | -0.33 | -0.06 |
| Problem solving (CSI) | -0.66 | 0.05 | -12.45 | 0.001 | -0.76 | -0.55 |
